# Detection and Characterization of Circulating Tumour Cells from Frozen Peripheral Blood Mononuclear Cells

**DOI:** 10.5772/60745

**Published:** 2015-05-13

**Authors:** David Lu, Ryon P. Graf, Melissa Harvey, Ravi A. Madan, Christopher Heery, Jennifer Marte, Sharon Beasley, Kwong Y. Tsang, Rachel Krupa, Jessica Louw, Justin Wahl, Natalee Bales, Mark Landers, Dena Marrinucci, Jeffrey Schlom, James L. Gulley, Ryan Dittamore

**Affiliations:** 1 Epic Sciences, Inc., San Diego, CA, USA; 2 National Cancer Institute, National Institutes of Health, Bethesda, MD, USA

**Keywords:** Circulating Tumour Cells, Peripheral Blood Mononuclear Cells, Metastatic Castrate Resistant Prostate Cancer, Androgen Receptor, Biorepository

## Abstract

Retrospective analysis of patient tumour samples is a cornerstone of clinical research. CTC biomarker characterization offers a non-invasive method to analyse patient samples. However, current CTC technologies require prospective blood collection, thereby reducing the ability to utilize archived clinical cohorts with long-term outcome data. We sought to investigate CTC recovery from frozen, archived patient PBMC pellets. Matched samples from both mCRPC patients and mock samples, which were prepared by spiking healthy donor blood with cultured prostate cancer cell line cells, were processed “fresh” via Epic CTC Platform or from “frozen” PBMC pellets. Samples were analysed for CTC enumeration and biomarker characterization via immunofluorescent (IF) biomarkers, fluorescence in-situ hybridization (FISH) and CTC morphology. In the frozen patient PMBC samples, the median CTC recovery was 18%, compared to the freshly processed blood. However, abundance and localization of cytokeratin (CK) and androgen receptor (AR) protein, as measured by IF, were largely concordant between the fresh and frozen CTCs. Furthermore, a FISH analysis of PTEN loss showed high concordance in fresh vs. frozen. The observed data indicate that CTC biomarker characterization from frozen archival samples is feasible and representative of prospectively collected samples.

## 1. Introduction

The molecular characterization of circulating tumour cells (CTCs) in the blood of patients with cancer has garnered great interest for its potential to longitudinally monitor an evolving disease, response to therapy and/or define prognosis [1][Bibr bibr2-60745][Bibr bibr3-60745][Bibr bibr4-60745]–[5]. While numerous CTC technologies are in development, a recognized unmet need is the ability to retrospectively analyse CTC samples from previously archived (frozen) clinical samples with associated long clinical histories [6].

The Epic CTC Platform utilizes a non-enrichment-based method and slide-based immunofluorescence (IF), coupled with digital pathology and genomic techniques, to detect and molecularly characterize CTCs. As part of the Epic Sciences standard operating procedures (SOPs), blood tubes are shipped to Epic Sciences and processed within 96 hours from blood draw. Following red blood cell (RBC) lysis, the nucleated fraction is plated onto microscope slides and frozen at −80°C for storage and subsequent analysis. In contrast, many enrichment-based strategies are unable to store morphologically intact CTCs for future analysis [7][Bibr bibr8-60745]–[9]. In order to augment the Epic SOP for sample processing, we sought to determine if the Epic Platform might be compatible with previously banked (frozen) patient material to enable retrospective analysis and expand the patient sample pool amenable to Epic CTC characterization.

The collection of patient tissue, blood, saliva and urine is common in clinical trials, often with peripheral blood mononuclear cells (PBMCs) subjected to isolation and cryopreservation [10]. Often, samples are banked until retrospective analyses are initiated. To enable the retrospective analysis of existing archived samples, we sought to test whether CTCs could be detected and molecularly characterized from pelleted, frozen PBMC fractions using the Epic CTC Platform. We then compared the CTCs recovered from these “frozen” samples with matched material, which was prepared “fresh” per Epic SOP.

In this study, we report the enumeration and biomarker characterization of spiked controls and patient samples, which were processed fresh at Epic Sciences. These are compared to matched samples from frozen/archived PBMCs, which were prepared by Ficoll separation. We also compare the morphological characteristics, protein expression and genetic alterations of CTCs that were processed using the Epic platform with CTCs from frozen PBMCs, which had been stored up to 7.5 years prior to analysis.

## 2. Material and Methods

### 2.1 Preparation of Control Cell Line Cell (CLC) Slides

Healthy donor (HD) blood was collected in sodium heparin Vacutainer® tubes (BD, Franklin Lakes, NJ) and whole blood white blood cell (WBC) counts were recorded. Known amounts of VCaP or PC3 (ATCC, Manassas, VA) prostate cancer cell line cells (CLCs) were spiked into the HD samples and nucleated cells were isolated by either the Epic SOP or Ficoll density separation (Ficoll-Paque; GE Healthcare, Buckinghamshire, UK), as per the manufacturer's protocol. For a description of the Epic SOP, please see “Analytical Validation and Capabilities of the Epic CTC Platform: Enrichment-Free Circulating Tumor Cell Detection Characterization” [11]. WBC/PBMC counts were taken after purification and were used to calculate the per cent recovery. The recovered cell line-spiked PBMC pellets were either: 1) used to create control CLC slides for IF staining per Epic SOP via cell deposition on microscope slides and storage in the Epic Biorepository, or 2) cryopreserved in 90/10 human AB serum/DMSO (Sigma-Aldrich, St. Louis, MO) and stored in liquid nitrogen for at least 48 hours. The cryopreserved PBMC pellets were then thawed and deposited onto glass slides for staining and analysis or storage in the Epic Biorepository.

### 2.2 Patient Blood Sample Processing

Blood samples were collected using Cell Free DNA BCT® tubes (Streck, Omaha, NE) from metastatic castrate resistant prostate cancer (mCRPC) patients at the National Cancer Institute and shipped to Epic Sciences for processing within 96 hours. All patients gave informed consent and enrolled on a biospecimen collection protocol approved by the NCI IRB (NCT00034216). Nucleated cells were deposited onto glass slides and subsequently stored at −80°C, as per Epic SOP. For matched patient blood samples, two identical blood tubes were drawn. One tube per patient was shipped immediately to Epic Sciences and processed via SOP, while trained operators processed the second tube at the clinical site via Ficoll separation. Ficoll purified PBMCs were cryopreserved in 90/10 human AB serum/DMSO and frozen in liquid nitrogen for up to 90 months prior to CTC analysis. Archived samples were shipped frozen to Epic Sciences and processed with a modified protocol for PBMC and CTC thaw and recovery, followed by immediate cell deposition onto slides and subsequent Epic Biorepository storage. For concordance testing, matched samples were stained and processed identically for morphology and biomarker assessment.

### 2.3 Cryopreserved CLC/CTC Thaw

For cryopreserved samples, the CLCs and patient samples, which were stored in liquid nitrogen, were thawed in a 37°C water bath, followed by an immediate dilution with RPMI 1640 medium supplemented with 10% foetal bovine serum (Life Tech, Carlsbad, CA). The PBMCs were pelleted and washed with PBS. This was followed by cell counting, resuspension in plating medium and deposition onto glass slides for −80°C storage and CTC analysis.

### 2.4 Immunofluorescent Staining and Analysis

Epic Sciences' CTC identification and characterization technology has been described in previous publications [11, 12]. In brief, slides created from CLC-spiked HD samples or mCRPC patient samples were subjected to automated immunofluorescent (IF) staining utilizing commercially available monoclonal primary antibodies against pan-cytokeratin (CK), CD45 and androgen receptor (AR). Fluorescent-labelled Alexa Fluor secondary antibodies (Life Tech, Carlsbad, CA) were used to allow for fluorescent detection and semi-quantitative characterization of protein biomarkers. The stained slides were analysed with automated fluorescent scanners and morphology algorithms for the identification of traditional (CK+) CTCs, CTC clusters, small CTCs, apoptotic cells and CK-negative (CK-) CTCs. A more thorough description of CTC types and their stage of validation is described in “Analytical Validation and Capabilities of the Epic CTC Platform: Enrichment-Free Circulating Tumor Cell Detection Characterization” [11]. Trained classifiers conduct a final classification of CTC subpopulations and record subcellular biomarker localization, where applicable.

#### 2.5 Fluorescence in Situ Hybridization

Following CTC enumeration, IF and morphological characterizations, select slides with sufficient CTCs were further tested for genetic alterations by fluorescence in situ hybridization (FISH). Coverslips were removed, immunofluorescence staining was attenuated and cells were fixed and dehydrated with formaldehyde and ethanol, respectively. After dehydration, a probe solution targeting the DNA sequences of interest (i.e., PTEN) was applied to each slide, denatured for 10 minutes at 83°C and hybridized for 14-24 hours at 37°C. The slides were washed in a series of saline sodium citrate (SSC)/detergent (Igepal) solutions, counterstained with DAPI and mounted with an anti-fade mounting medium. As the exact coordinates of every CTC were recorded during the enumeration process, each CTC can be relocated and analysed by FISH for specific genetic alterations. Patient WBCs were used as internal controls, representing the normal genetic status for each respective sample.

#### 2.6 Statistical Analyses

Statistical analyses were conducted using Graphpad Prism software (La Jolla, CA). Kruskal-Wallis ANOVA was used to compare the measures with >2 groups. A Mann-Whitney U test was used to compare the differences between the matched sample pairs from each individual patient. P<0.05 was considered to be significant.

### 3. Results

#### 3.1 Androgen Receptor (AR)+ and AR- Cell Lines Demonstrate Feasibility of CTC Recovery from Cryopreservation

**Figure 1.**
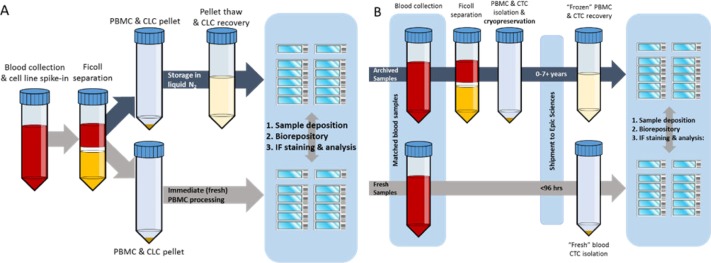
Schematic of sample preparation and experimental protocol for fresh and frozen sample processing. **(A)** To create control slides, cell line cells (CLCs) were spiked into healthy donor blood and processed via Ficoll separation. CLC-containing PBMC pellets were split into identical fractions: one fraction was cryopreserved in liquid nitrogen prior to cell deposition (blue arrow), and the other fraction was deposited fresh onto slides (grey arrow). **(B)** Matched mCRPC patient samples were drawn: one sample was sent immediately to Epic Sciences for fresh sample deposition (grey arrow), and one sample was processed via Ficoll and frozen at the clinical site. The frozen PBMC sample was cryopreserved for up to 7.5 years prior to shipment and subsequent processing at Epic Sciences (blue arrow).

**Figure 2.**
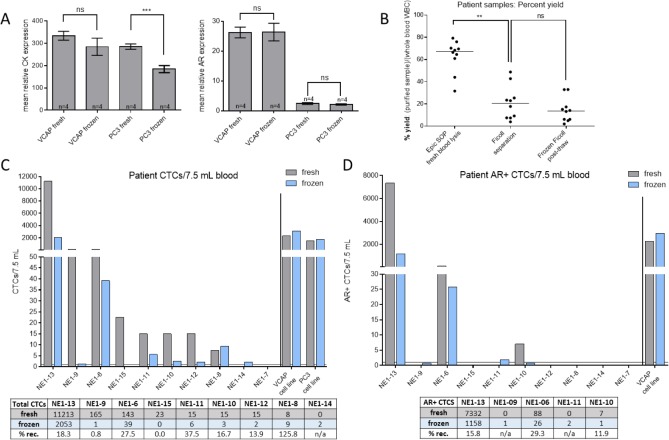
Assessment of cell line and mCRPC patient CTC recovery. **(A)** The mean CLC CK and AR protein expression levels were compared between cells that were processed fresh and after cryopreservation and thaw. The cryopreserved VCaP and PC3 samples showed a mean decrease in CK expression of 15% (p>0.05) and 35% (p<0.001), respectively, compared to freshly processed cells. Mean VCaP and PC3 AR expression remained unchanged. **(B)** Compared to whole blood WBC counts, the median WBC recovery in the patient samples that were processed fresh per Epic SOP was 67% (range: 32-79), while Ficoll purification resulted in a median PBMC yield of 20% (range: 3.9-49). Ficoll-processed frozen sample recovery was not significantly changed post-thaw (p>0.05). The total CTCs **(C)** and AR+ CTCs detected **(D)** from matched mCRPC patient blood draws, which were normalized to 7.5 mL blood, are plotted and tabulated alongside control PC3 and VCaP CLC control slides. Patient CTCs were found in 8/10 of the fresh samples and 8/10 of the frozen samples. Compared to the fresh samples, the median total and AR+ CTC recovery in the frozen samples were 18% and 16%, respectively. Notable differences: CTCs were found in the fresh sample from patient NE1-15 but were undetected in the matched frozen sample, while CTCs were found in the frozen NE1-14 sample, which went undetected in the fresh material. **p<0.01, ***p<0.001, n=10 patient samples.

To initially assess the capabilities of frozen CTC recovery on the Epic Platform, CTC surrogate (VCaP (AR+) and PC3 (AR-)) CLCs were spiked into HD blood and subjected to the same cell isolation, cryopreservation and sample deposition protocols to be used for the processing of archived patient samples (Figure 1A). Matched slides containing CLCs that were deposited fresh or after freezing, were stained for CK, CD45 and AR. The concordance of PBMC and CLC morphology, as well as protein expression, were compared (Figure 2A). No statistically significant differences in recovery rates were observed between CLCs that were processed with or without cryopreservation (not shown). No significant changes in the mean AR expression were seen in either VCaP or PC3 cells in frozen samples, indicating the preservation of AR protein integrity. VCaP CK expression also remained unchanged, while PC3 CK expression decreased by 35% in the frozen samples (p<0.01). However, in the latter case, relative CK staining intensity was still observed at 180-fold over WBC background.

#### 3.2 Concordance of CTC Recovery and Protein Expression from Archived mCRPC Patient Samples

To determine concordance of patient CTC recovery and characterization, two matched blood tubes were drawn from 10 mCRPC patients. One tube was shipped to Epic Sciences and processed within 96 hours using standard cell deposition protocols (fresh), while the second tube was subject to Ficoll PBMC isolation and archived in liquid nitrogen (frozen; Figure 1B). Compared to the fresh samples that were undergoing RBC lysis at Epic Sciences, the Ficoll separation resulted in a lower net yield of nucleated cells due to the preferential enrichment for PBMC over the whole WBC (median recovery: 67% and 20%, respectively; Figure 2B). Notably, the subsequent freeze/thaw of the Ficoll isolated samples had no significant effect on the net PBMC recovery (p>0.05), indicating that the freeze/thaw process lost very little patient material.

**Figure 3.**
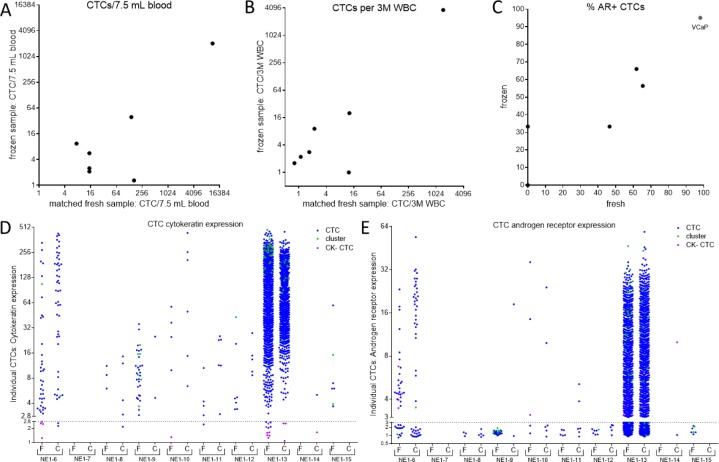
Comparison of CTC enumeration and biomarker expression. The CTCs that were recovered from the matched samples (or VCaP control) show a linear correlation between the fresh and cryopreserved preparations when normalized to either 7.5 mL blood **(A),** 3M WBC **(B),** or **(C)** per cent AR+ CTCs (r^2^≥0.90 for each comparison). CTCs with heterogeneous CK **(D)** and AR **(E)** expression levels were detected in the majority of patients from both the fresh (F) and cryopreserved (C) samples. The dot plot depicts the presence of individual CTCs found in each patient sample. CK and AR positivity was defined for any CTC with a relative protein expression of greater than 2.8- or 3-fold over background, respectively (dotted lines). With the exception of NE1-6 CK expression (**D**; p<0.01), no other matched patient samples expressed significantly different levels of mean CK or AR protein between the fresh and frozen material (p>0.05; Mann-Whitney U test).

The total CTCs and AR+ CTCs, as detected by IF staining, were normalized to 7.5 mL blood volume and compared between the freshly processed and archived samples (Figure 2C, D). CTCs were found in 8/10 of the fresh samples and 8/10 of the frozen archived samples. Typical CTC recovery in frozen samples was significantly lower as median CTC recovery in frozen samples was 18% that observed from freshly processed blood. However, the relative order of CTC burden in patients remained fairly consistent, with the exception of two patients, NE1-8, −9. Furthermore, CTCs were found in the fresh sample from patient NE1-15, which were undetected in the matched frozen sample. Meanwhile, CTCs were found in the frozen NE1-14 sample, which went undetected in the corresponding fresh material. AR+ CTCs were found in both preparations of NE1-13, −6 and −10, and the median recovery of AR+ CTCs from the frozen samples was 16% of that observed in freshly processed blood. Notably, the CTC count correlates linearly (though sample size is limited) between the fresh and frozen samples when normalized to blood volume or WBC count (r^2^>0.90, n=10; Figure 3A, B). Additionally, the per cent of AR+ CTC/CLC subpopulations also correlate between the matched samples (r^2^=0.90, n=10; Figure 3C).

#### 3.3 Cytokeratin and Androgen Receptor Expression is Retained in Cryopreserved CTCs

**Figure 4.**
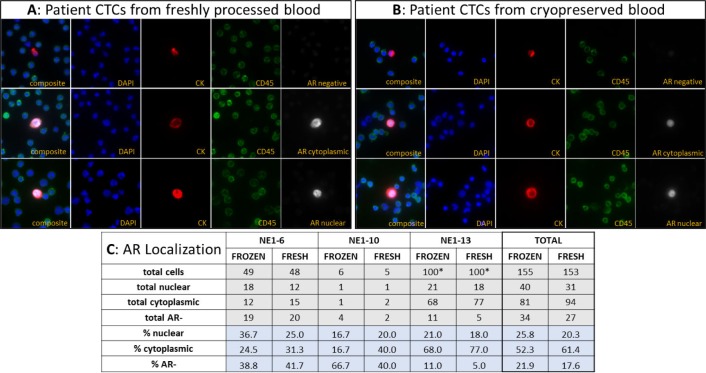
Representative images of patient CTCs demonstrate the preservation of WBC and CTC morphology and retention of AR localization. Of the eight patients with detectable CTCs, three patients harboured AR+ CTCs in both fresh and frozen samples. Representative images of AR- CTCs, nuclear AR+ CTCs and diffuse cytoplasmic AR+ CTCs, which were recovered from both the fresh **(A)** and frozen **(B)** samples, are shown depicting preserved WBC and CTC morphology and consistent biomarker localization. **(C)** Numerical assessment of AR localization in AR+ patients confirms the retention of subcellular protein localization after CTC cryopreservation. The percentages of CTCs with nuclear or cytoplasmic localized AR are similar between the matched AR+ samples, with no significant bias of localization between the fresh and frozen samples. (*>1300 CTCs present per slide; only 100 CTCs were assessed for AR localization.)

Heterogeneous CK and AR protein expression, as quantified by IF staining, was observed in all of the CTC + patients (Figure 3D, E). Moreover, traditional CK+ CTCs, CTC clusters and CK- CTC subpopulations were found in both the fresh and frozen material. With the exception of NE1-6, in which the median CK expression was higher in the cryopreserved sample (p<0.01), no other significant differences in CK or AR protein levels were observed between the matched material. Furthermore, within the AR+ CTCs, subpopulations with predominately nuclear-localized AR as well as CTCs with diffuse cytoplasmic AR were detected (representative images, Figure 4A, B). No notable differences in the distribution of AR localization were observed in the AR+ CTC subpopulations. (Figure 4C). Thus, the process of cryopreservation did not disrupt the AR or CK protein localization in these samples.

#### 3.4 Concordance of Genetic Assessment by FISH

**Figure 5.**
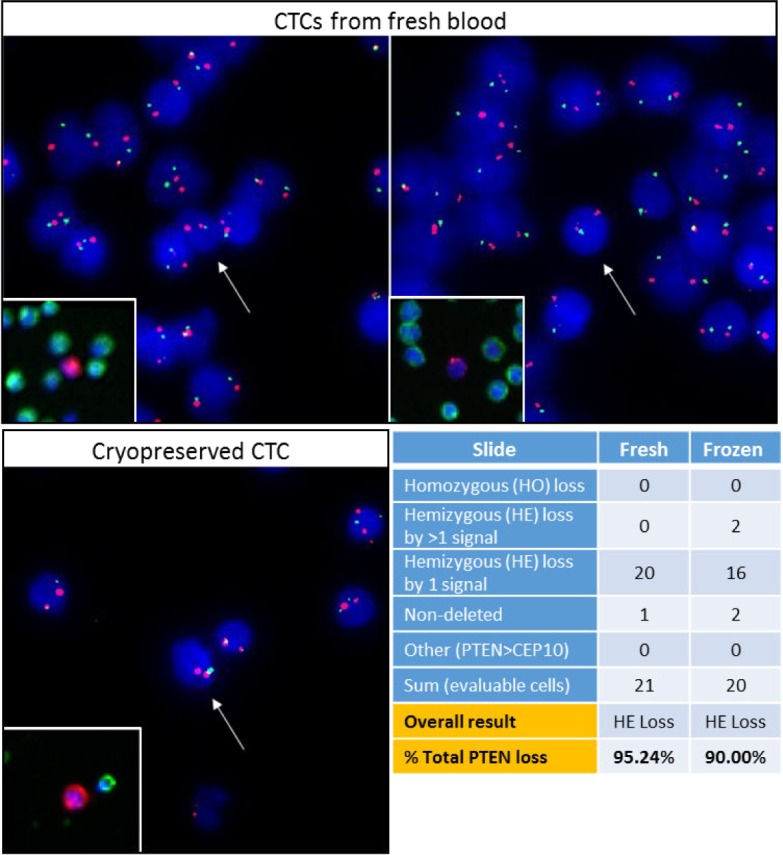
Cryopreserved CTCs from PBMC pellets are viable for genetic analysis. After IF staining and protein characterization, patient CTCs recovered from cryopreservation were assessed via PTEN FISH. CTC homozygous PTEN loss is defined as the complete absence of PTEN (10q23.31; green) signals with the presence of ≥1 centromeric CEP10 (10p11.1-q11.1; red) signal(s) within the same cell. CTCs with present PTEN signals < CEP10 signals are indicative of hemizygous PTEN loss. Preservation of WBC and CTC morphology was observed in frozen samples after fluorescent probe hybridization as well as a consistent assessment of PTEN hemizygous loss in all CTCs assessed (95% & 90% PTEN loss in fresh and frozen material, respectively). Neighboring WBCs were used as internal controls for normal genetic status and to establish proper assay specificity.

To test the viability of downstream genetic analyses on cryopreserved CTCs, PTEN FISH was conducted on patient CTCs detected from freshly processed blood (Epic SOP) and Ficoll-processed cryopreserved samples. In each sample from patient NE1-13, at least 20 CTCs were assessed for PTEN alterations. The assessment of homozygous (PTEN = 0, CEP10 ≥ 1) and hemizygous (0 < PTEN < CEP10) PTEN loss found near-equivalent populations of genetic alterations between the fresh and frozen CTCs (Figure 5). These findings demonstrate the preservation of DNA and the feasibility of genetic analyses in cryopreserved CTCs.

#### 3.5 CTC Recovery from Additional Banked mCRPC Patient Samples

**Figure 6.**
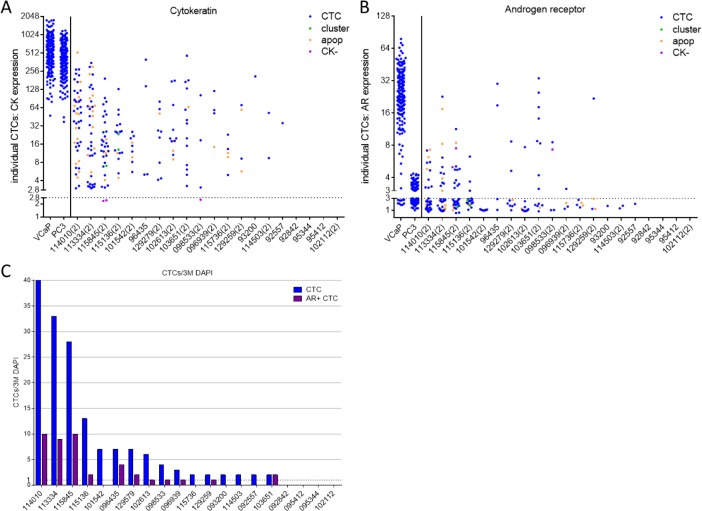
Additional cryopreserved patient samples were processed via the Epic CTC Platform. Twenty additional cryopreserved mCRPC patient samples of up to 7.5 years of age were processed. Similar to previously characterized matched samples, IF staining detected CK+ CTCs, CTC clusters, CK- CTCs and apoptotic cells with heterogeneous CK **(A)** and AR **(B)** expression. The threshold for CK and AR positivity is 2.8- and 3-fold over background signals, respectively (dotted lines). **(C)** Out of 20 samples, 16 (80%) had detectible CTCs (range: 2-40 CTCs/3M WBCs) and 11/16 (69%) CTC+ samples included AR+CTCs (range: 1-10 CTCs/3M WBCs).

Cryopreserved mCRPC patient PBMC samples from a previously completed clinical trial were shipped to Epic Sciences for CTC recovery and characterization (5-92 months old). CTCs were detected in 16 of 20 (80%) samples, with 11 patients also harbouring AR+ CTCs (Figure 6). Akin to previously analysed samples, CTCs of varying morphology (i.e., traditional CK+, CTC clusters, apoptotic CTCs and CK- CTCs) were found. Furthermore, heterogeneous CK and AR protein levels and AR localization patterns were observed in CTCs from these samples.

## 4. Discussion

In this study, we report enumeration and characterization of morphology, biomarker expression, and genetic alterations of CTCs isolated from frozen PBMC fractions stored up to 7.5 years prior to analysis. We found that patient blood samples processed by Ficoll separation and cryopreserved in liquid nitrogen are amenable to CTC recovery and characterization using the Epic Platform. The Ficoll gradient method enriches for PBMCs, removing granulocytes and thus accounting for the decreased apparent yield of nucleated cells after processing. Compared to the Epic Sciences standard RBC lysis, this in part may account for the decreased CTC recovery in samples processed by Ficoll. However, the procedure of cryopreservation and sample thaw alone does not substantially alter cell recovery, indicating that the pre-analytic phase of nucleated cell isolation may play a large role in influencing WBC/PBMC (and presumably, CTC) recovery rates.

On average, approximately one quarter of the total CTC counts were detected when the PBMCs were isolated via Ficoll and frozen, as compared to the matched blood tubes, which were sent immediately to Epic and banked fresh per the Epic SOP. However, though absolute CTC recovery diminishes in the archived samples, the relative abundance of patient CTC burden, as well as the protein biomarker (CK, AR) expression of those CTCs, remains largely consistent with the matched fresh samples. In the case of the archived samples, a decreased net CTC recovery can potentially be overcome by testing a larger volume of blood. Importantly, the archived CTCs demonstrate preserved morphology, genetic alterations and biomarker localization as compared to the fresh samples that were received and processed within 96 hours. In our initial feasibility study with matched mCRPC patient samples, we were able to assess CTC CK and AR protein expression, AR localization and PTEN status, all of which were found to be largely concordant between the fresh and frozen material. Taken together, all of the CTCs across all of the frozen samples had a mean magnitude of CK and AR protein expression 90% and 107% of matched freshly processed material. Correlating these metrics between the matched samples shows that the process described does not introduce a significant bias towards any given cell population. Furthermore, no particular CTC subpopulation appears to be favoured by cryopreservation. These findings suggest that while precise longitudinal studies focused on CTC enumeration may not be accurate given pre-analytic variability (though relative abundance may be feasible), genetic and protein characterization of archived CTCs is possible on the Epic CTC Platform, enabling retrospective biomarker validation studies in large cohorts of patients with detailed clinical history.

In addition to these concordance studies, CTCs were detected in an additional 16 of 20 advanced mCRPC samples that were banked for up to 7.5 years prior to analysis. Notably, no correlation was seen between sample age and CTC recovery, and CTCs were found in both baseline and follow-up draws. The data presented here focus on the feasibility of studies based on archived samples and do not themselves constitute a comprehensive validation. Further work is underway to relate our findings with clinical data and assess the ability to prognosticate patient outcome based on data garnered from cryopreserved CTC analysis. Additionally, while we have observed close concordance of genetic PTEN alterations between fresh and frozen CTCs from the same patient, ongoing studies are working to relate CTC genetic alterations with incidence of mutations in both primary and metastatic tumour tissue. A manuscript describing close concordance of PTEN status between mCRPC CTCs characterized on the Epic platform and solid tumour biopsy tissue is currently under review for publication. Furthermore, the feasibility of single cell picking on the Epic CTC Platform coupled to next generation sequencing (NGS) has been demonstrated as a proof of concept (manuscript in preparation), and in-depth NGS characterization of archived patient CTCs is underway.

The ability to retrospectively analyse samples from completed prospective studies might yield additional CTC biomarkers or insights into drug response or resistance when more time-related clinical information, like PFS and OS, are available. Retrospective genomic analyses have been reported on “exceptional responder” patient samples to define the likely mechanism of response to targeted therapies [13, 14]. The ability to detect and characterize material from archived patient blood samples offers the potential for an additional window to augment archived solid tissue biopsies for such analyses.

## 5. Compliance with Ethical Research Standards

All of the patients gave informed consent and enrolled on a biospecimen collection protocol, approved by the NCI IRB (NCT00034216).

## 6. Disclosures

DL, RPG, MH, SB, RK, JL, JW, NB, ML, DM and RD are employees of Epic Sciences, Inc., San Diego, CA. All other authors declare no conflicts of interest.
